# Survival outcomes including salvage therapy of adult head and neck para-meningeal rhabdomyosarcoma: a multicenter retrospective study from Japan

**DOI:** 10.1186/s12885-023-11528-4

**Published:** 2023-10-31

**Authors:** Kenji Tsuchihashi, Mamoru Ito, Shuji Arita, Hitoshi Kusaba, Wataru Kusano, Takashi Matsumura, Takafumi Kitazono, Shohei Ueno, Ryosuke Taguchi, Tomoyasu Yoshihiro, Yasuhiro Doi, Kohei Arimizu, Hirofumi Ohmura, Tatsuhiro Kajitani, Kenta Nio, Michitaka Nakano, Kotoe Oshima, Shingo Tamura, Tsuyoshi Shirakawa, Hozumi Shimokawa, Keita Uchino, Fumiyasu Hanamura, Yuta Okumura, Masato Komoda, Taichi Isobe, Hiroshi Ariyama, Taito Esaki, Kazuki Hashimoto, Noritaka Komune, Mioko Matsuo, Keiji Matsumoto, Kaori Asai, Tadamasa Yoshitake, Hidetaka Yamamoto, Yoshinao Oda, Koichi Akashi, Eishi Baba

**Affiliations:** 1 Department of Hematology, Oncology and Cardiovascular Medicine, Kyushu University Hospital, Fukuoka, Japan; 2 Department of Medical Oncology Organization, Hamanomachi Hospital, Fukuoka, Japan; 3 Department of Medicine and Biosystemic Science, Graduate School of Medical Sciences, Kyushu University, Fukuoka, Japan; 4 Department of Medical Oncology, Clinical Research Institute, National Hospital Organization Kyushu Medical Center, Fukuoka, Japan; 5 Department of Hematology and Oncology, Japan Community Health care Organization Kyushu Hospital, Fukuoka, Japan; 6 Department of Gastrointestinal and Medical Oncology, National Hospital Organization Kyushu Cancer Center, Fukuoka, Japan; 7 Department of Internal Medicine, Kyushu University Beppu Hospital, Oita, Japan; 8 Department of Oncology and Social Medicine, Graduate School of Medical Sciences, Kyushu University, 3-1-1 Maidashi, Fukuoka, Higashi-ku 812-8582 Japan; 9 Department of Otorhinolaryngology, Graduate School of Medical Sciences, Kyushu University, Fukuoka, Japan; 10 Department of Clinical Radiology, Graduate School of Medical Sciences, Kyushu University, Fukuoka, Japan; 11 Department of radiation therapy, Hamanomachi Hospital, Fukuoka, Japan; 12 Department of Anatomic Pathology, Graduate School of Medical Sciences, Kyushu University, Fukuoka, Japan

**Keywords:** Rhabdomyosarcoma, Adult, Head and neck, Para-meningeal, Salvage

## Abstract

**Background:**

Rhabdomyosarcoma is the most common soft tissue sarcoma in children, but rare in adults. Para-meningeal rhabdomyosarcoma in head and neck (PM-HNRMS) is less applicable for surgery due to the anatomic reason. PM-HNRMS has a poor prognosis in children. However, its clinical outcomes remain unclear in adults due to the rarity. Further, there is almost no detailed data about salvage therapy.

**Methods:**

We retrospectively examined the adult patients with PM-HNRMS treated at institutions belonging to the Kyushu Medical Oncology Group from 2009 to 2022. We evaluated the overall survival (OS) and progression-free survival (PFS) of the patients who received a first-line therapy. We also reviewed the clinical outcomes of patients who progressed against a first-line therapy and received salvage therapy.

**Results:**

Total 11 patients of PM-HNRMS received a first-line therapy. The characteristics were as follows: median age: 38 years (range 25 – 63 years), histology (alveolar/spindle): 10/1, and risk group (intermediate/high): 7/4. As a first-line therapy, VAC and ARST0431-based regimen was performed in 10 and 1 patients, respectively. During a first-line therapy, definitive radiation for all lesions were performed in seven patients. The median PFS was 14.2 months (95%CI: 6.0 – 25.8 months): 17.1 months (95%CI: 6.0 – not reached (NR)) for patients with stage I-III and 8.5 months (95%CI: 5.2 – 25.8 months) for patients with stage IV. The 1-year and 3-year PFS rates were 54.5% and 11.3% for all patients. Median OS in all patients was 40.8 months (95%CI: 12.1 months–NR): 40.8 months (95%CI: 12.1 – NR) for patients with stage I-III and NR for patients with stage IV. The 5-year OS rate was 48.5% for all patients. Among seven patients who received salvage therapy, three are still alive, two of whom remain disease-free for over 4 years after completion of the last therapy. Those two patients received multi-modal therapy including local therapy for all detected lesions.

**Conclusion:**

The cure rate of adult PM-HNRMS is low in spite of a first-line therapy in this study. Salvage therapy might prolong the survival in patients who received the multi-modal therapy including local therapy for all detected lesions.

## Introduction

Rhabdomyosarcoma (RMS) is the most common soft tissue sarcoma in children, accounting for more than 50% of soft tissue sarcoma cases [1], whereas RMS in adults is rare because its cases are fewer than 4% of adult soft tissue sarcomas [2]. The prognosis of adult RMS is worse than that of children [1]. This is considered to be due to differences in pathogenesis, histology subtype, primary location and applied multimodality treatment between children and adults [1]. Primary head and neck RMS (HNRMS) accounts for 9.3% of adult RMS cases. Location in head and neck RMS is divided into orbital, para-meningeal (PM) and non-para-meningeal (non-PM) sites [3]. PM sites are nasopharynx, nasal cavity, parapharyngeal area, paranasal sinuses, infratemporal and pterygopalatine fossa, middle ear, mastoid and other sites extending to the PM region [4]. The incidence of PM and non-PM HNRMS is almost equal and orbital sites are less than those [1].

PM-HNRMS is less applicable for surgery compared with non-PM-HNRMS due to anatomical reasons. The Intergroup Rhabdomyosarcoma Study (IRS) suggested a post-surgical classification of RMS based on the extent of its surgical excision. According to this classification, group III indicates localized tumor with gross residual disease after incomplete resection or biopsy alone. In localized non-PM-HNRMS patients, 78% of patients were categorized as IRS post-surgical classification group III [3]. On the other hand, 95% of PM-HNRMS patients were classified as group III [5]. Additionally, 60.6% cases of orbital RMS show localized disease, while 53.2% of PM-HNRMS cases show regional disease and 28.1% have distant spread [6]. In children, the 5-year overall survival (OS) of non-PM-HNRMS and PM-HNRMS in non-metastatic patients was reported to be 87% and 71%, respectively [7]. However, in adult cases, there are only few previous reports on PM-HNRMS, and hence, more studies are necessary to clarify its outcomes [8].

In the present study, we retrospectively investigated the clinical outcomes of adult PM-HNRMS patients.

### Patients

We retrospectively reviewed the medical records of consecutive patients with adult PM-HNRMS patients treated at institutions belonging to the Kyushu Medical Oncology Group from 2009 to 2022. The cutoff date was July 2023. The eligibility criteria for study inclusion were: age ≥ 18 years, histologically proven RMS in the PM head and neck region, and receiving chemotherapy as first-line therapy. PM sites were defined as the nasopharynx, nasal cavity, parapharyngeal area, paranasal sinuses, infratemporal and pterygopalatine fossa, middle ear, and mastoid and other sites extending to PM sites. Detection of fusion gene was conducted by polymerase chain reaction (PCR).

### Treatments

As a first-line chemotherapy, VAC and ARST-0431 based regimen was performed. VAC regimen is as follows: vincristine 1.5 mg/m^2^ on days 1, 8 and 15, actinomycin D 0.045 mg/kg on day 1, cyclophosphamide 1,500 mg/m^2^ – 2,200 mg/m^2^ on day 1, with the cycle repeated every 3 weeks) for a total of 14 cycles [9]. ARST0431 regimen, comprising of VI (vincristine and irinotecan), VDC/IE (vincristine, doxorubicin and cyclophosphamide/ifosfamide and etoposide) and VAC, is performed following the previous study with some modification [10].

Salvage chemotherapy included a regimen based on ARST0121, comprising of VI, DC (doxorubicin and cyclophosphamide) and IE [11], ICE regimen (ifosfamide 1,800 mg/m^2^ on days 1–5, carboplatin 400 mg/m^2^ on days 1,2 and etoposide 100 mg/m^2^ on days 1–5, repeated every 3 weeks), and reintroduction of VAC. IE was also performed as salvage chemotherapy at another hospital after the patient was transferred.

Radiotherapy for all lesions were performed with the total 50.4 Gy in cases with stage I – III disease. Radiotherapy for limited lesions indicates radiation with 50.4 Gy for the local lesions in cases with stage IV RMS. One patient received carbon ion radiotherapy with 46.8 Gy (relative biological effectiveness) for limited lesion. Prophylactic irradiation for regional lymph nodes were not performed when primary site was irradiated.

### Assessment

Evaluation of tumor lesions was conducted by computed tomography (CT) scans, which was basically planned every 2–3 months. CT was performed earlier in cases with worsening subjective symptoms. Progression-free survival (PFS) was calculated from the date of administration of first-line chemotherapy to the date of progression or death from any cause, whichever was earlier, or was censored at the final follow-up examination. OS was defined as the period from the initiation of first-line chemotherapy to the day of death from any cause or was censored at the final follow-up examination. Tumor responses were assessed using the Response Evaluation Criteria in Solid Tumors (RECIST) version 1.1.

### Statistical analysis

PFS and OS were estimated by the Kaplan–Meier method. All statistical analyses were performed using JMP software (SAS Institute Japan, Tokyo, Japan).

## Results

### Patients’ characteristics

**Fig. 1:**
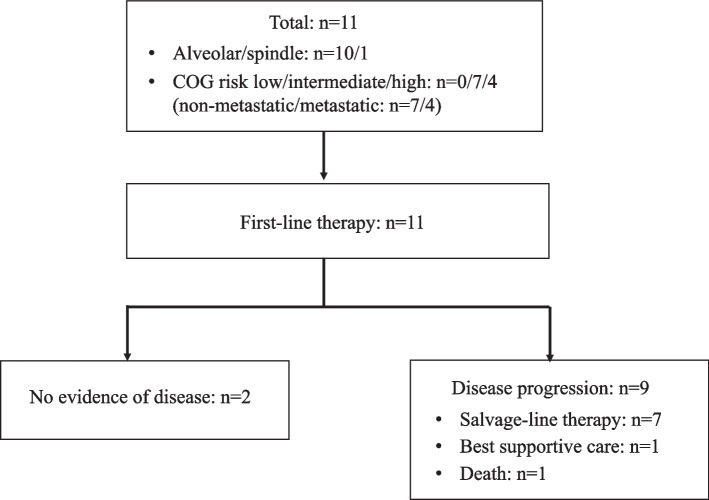
Flow diagram of the present study

Total eleven patients were included as shown in Fig. 1. Patient characteristics are shown in Table 1. The median follow-up period from the date of administration of first-line chemotherapy was 6.1 year (range 1.4–13.8 years). Seven females and four males were examined. Median patient age was 38 years (range 25–63 years). Ten and one patients had alveolar and spindle histology, respectively. *PAX3-FOXO1* fusion was detected in nine of ten patients with the alveolar subtype. One patient with alveolar subtype was negative for both *PAX3-FOXO1* and *PAX7-FOXO1* fusion. *MyoD1* mutation was detected in the patient with spindle histology. In terms of the Children’s Oncology Group (COG)-risk group, seven and four patients were considered intermediate and high risk, respectively. There were no patients with the history of head and neck cancer.

**Table 1:** Patients’ characteristics

	Total (*n* = 11)
Median age (range)	38 (25–63)
Gender, n (%)	
Female	7 (64)
Male	4 (36)
Primary lesion, n (%)	
Paranasal sinus	7 (64)
Nasal cavity	3 (27)
Pterygopalatine fossa	1 (9)
Histological type, n (%)	
Alveolar	10 (91)
Spindle	1 (9)
Gene alteration, n (%)	
*PAX3-FOXO1* fusion	9 (82)
*MyoD1* mutation	1 (9)
No	1 (9)
Tumor size, n (%)	
< 5cm	8 (73)
≥ 5cm	3 (27)
Reginal lymph node metastasis, n (%)	
No	6 (55)
Yes	5 (45)
Distant metastasis, n (%)	
No	7 (64)
Yes	4 (36)
TNM, n (%)	
1/2/3/4	1(9)/2(18)/4(36)/4(36)
Clinical group, n (%)	
I/II/III/IV	1(9)/0(0)/6(55)/4(36)
COG risk, n (%)	
Low/intermediate/high	0(0)/7(64)/4(36)

### First-line treatment and outcome

**Fig. 2:**
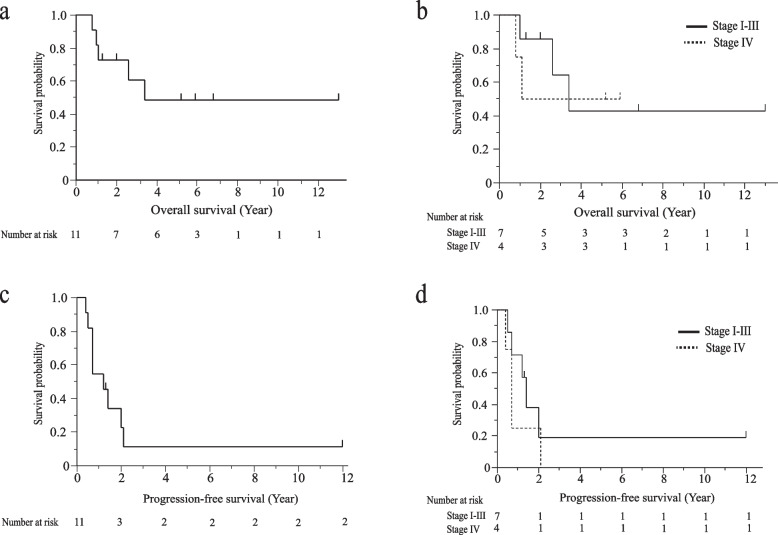
**a**. Kaplan–Meier plot for overall survival of all patients. **b**. Kaplan–Meier plot for overall survival of stage I-III and stage IV patients. **c**. Kaplan–Meier plot for progression-free survival of all patients. **d**. Kaplan–Meier plot for progression-free survival of stage I-III and stage IV patients

As a first-line treatment, ten patients received VAC and the other received ARST0431-based regimens as shown in Table 2. Eight of the ten patients treated with VAC started cyclophosphamide at a dose of 2,200 mg/m^2^, and one and another patient started with a dose of 1,500 mg/m^2^ and 1,650 mg/m^2^, respectively. The reasons for dose reduction in the two patients were considering the risk of infection due to severe neutropenia and old age. In nine of ten patients who received the VAC regimen except for one patient who moved to other hospital in the middle of treatment, the median cumulative cyclophosphamide dose was 20.7 g/m^2^ (range 6.6–30.8). Median administration frequencies of vincristine and actinomycin D were 23 times (range 7–36) and 11 times (range 4–12), respectively. Radiotherapy for all lesions and limited lesions was performed in six and three patients, respectively. In patients who received radiotherapy for limited lesion, carbon ion radiotherapy was performed in patient No.2. Eight patients completed a first-line therapy. Two patients progressed during first-line therapy. One patient stopped first-line therapy because of her request and experienced the progression. There was no therapy-related death. Median OS was 40.8 months (95%CI 12.1 months–NR) for all patients, 40.8 months (95%CI 12.1 months- not reached (NR)) for patients with stage I-III and NR for patients with stage IV as shown in Fig. 2a and b. The 5-year OS rate was 48.5% for all patients, 42.8% for patients with stage I-III and 50% for patients with stage IV. Median PFS was 14.2 months (95%CI 6.0–25.8 months) for all patients, 17.1 months (95%CI: 6.0 – NR) for patients with stage I-III and 8.5 months (95%CI: 5.2 – 25.8 months) for patients with stage IV as shown in Fig. 2c and d. The 1-year and 3-year PFS rates were 54.5% and 11.3% for all patients, 71.4% and 19.1% for patients with stage I-III, and 25% and 0% for patients with stage IV. Overall response rate for chemotherapy until the initiation of radiotherapy was assessed in eight patients with measurable lesions. Overall response rate was 100%. All were partial response.

**Table 2:** Patients’ characteristics and clinical courses

Pt	Age	Sex	Primary site	Histology	Gene alteration	T	N	M	Stage	Clinical group	COG risk	First-line chemo- therapy	Radiation therapy	Progression	Progression site	Salvage-line therapy	Present Status
1	25	F	Paranasal	Alveolar	*PAX3-FOXO1*	T2b	N1	M0	3	III	Inter-mediate	VAC	No (refused)	Yes	Primary, RL	Radiation, VAC	DOD
2	28	M	Nasal	Alveolar	*PAX3-FOXO1*	T2b	N1	M0	3	III	Inter-mediate	VAC	Limited lesion (carbon ion radiotherapy)	Yes	Meningeal dissemination	ICE	DOD
3	29	M	Paranasal	Alveolar	*PAX3-FOXO1*	T2a	N1	M0	3	III	Inter-mediate	VAC	All lesions	No	-	-	NED
4	34	F	Paranasal	Alveolar	*PAX3-FOXO1*	T2b	N0	M0	2	III	Inter-mediate	VAC	All lesion	No	-	-	NED
5	34	F	Paranasal	Alveolar	*PAX3-FOXO1*	T2a	N0	M1	4	IV	High	VAC	All lesions	Yes	Lung, DL	Radiation, ARST0121- based	NED
6	38	M	Pterygo-palatine fossa	Spindle	*MyoD1*	T2b	N0	M0	3	III	Inter-mediate	VAC	All lesion	Yes	Pleural dissemination	-	AWD
7	40	F	Paranasal	Alveolar	Not detected	T2b	N1	M1	4	IV	High	ARST0431-based	Limited lesion	Yes	Meningeal dissemination	-	DOD
8	41	M	Paranasal	Alveolar	*PAX3-FOXO1*	T2a	N0	M1	4	IV	High	VAC	Limited lesion	Yes	Bone	Radiation, IE	DOD
9	48	F	Nasal	Alveolar	*PAX3-FOXO1*	T2a	N1	M1	4	IV	High	VAC	All lesions	Yes	Breast	Surgery, IE	AWD
10	59	F	Nasal	Alveolar	*PAX3-FOXO1*	T1a	N0	M0	1	I	Inter-mediate	VAC	No	Yes	Primary, RL	Surgery, radiation, ARST0121- based	NED
11	63	F	Paranasal	Alveolar	*PAX3-FOXO1*	T2a	N0	M0	2	III	Inter-mediate	VAC	All lesions	Yes	RL	Surgery, VAC	DOD

*Pt* patient, *VAC* vincristine, actinomycin D and cyclophosphamide, *ARST0431* VI (vincristine and irinotecan), VDC/IE (vincristine, doxorubicin and cyclophosphamide/ifosfamide and etoposide) and VAC, *RL* regional lymph node, *DL* distant lymph node, *ICE* ifosfamide, carboplatin and etoposide, *ARST0121* VI, DC (doxorubicin and cyclophosphamide) and IE, *DOD* died of disease, *NED* no evidence of disease, *AWD* alive with disease

### Salvage-line treatment and outcome

Two and seven patients progressed during first-line therapy and during observational period after the completion of first-line therapy, respectively. The sites of progression in these nine patients were: primary site relapse and regional lymph nodes in two patients, meningeal dissemination in two patients, regional lymph nodes in one patient, bone in one patient, lung and mediastinal lymph node in one patient, breast in one patient and pleural dissemination in one patient as shown in Table 2. Two patients who experienced the relapse at the primary site did not receive radiation or carbon ion radiotherapy for primary sites at the first-line therapy. Seven among the nine patients with progressive disease including one patient (No. 1) who stopped first-line therapy because of her request and experienced disease progression received salvage chemotherapy. Among two patients who did not receive salvage therapy, one patient (No. 7) died during first-line therapy due to meningeal dissemination of disease. Another patient (No. 6) who are positive for *MyoD1* mutation chose best supportive care. His follow-up period after relapse is still short. Salvage chemotherapy was started with an ARST0121-based regimen in two patients, VAC regimen in two patients, IE regimen in two patient and ICE regimen in one patient. Three (Nos. 5, 9, 10) of the seven patients were alive at the time of this study. Among them, two patients (Nos. 5, 10) who started with an ARST0121-based regimen combined with local therapy currently remain disease-free for over 4 years after completion of the last therapy and one patient continues to receive further chemotherapy for persistent disease. There was no therapy-related death. 

The detailed clinical courses of patient No.5 and No.10 was shown as follows. Patient No.5 experienced the relapse of one mediastinal lymph node and one left lung metastasis after 16.6 months from the last course of a first-line VAC therapy. She started receiving ARST0121 regimen. After three months from the initiation of ARST0121 regimen, radiotherapy with 50.4 Gy for lymph node and lung metastases are performed. During radiation therapy, we continued VI regimen (vincristine 1.5 mg/m^2^/day on days 1,8 and irinotecan 20 mg/m^2^/day on days 1–5, 8–12, repeated every 3 weeks). However, from the second course of VI regimen during radiotherapy, we omitted irinotecan because of considering the incident risk of pneumonitis by continuing the combination of irinotecan and radiation. She completed all courses of ARST0121 regimen and keeps disease free for 4.4 years after the completion of salvage-line therapy. Patient No.10 had a relapse at the cervical lymph node after 7.2 months from the last course of a first-line VAC regimen. Firstly, she received the left cervical lymphadenectomy and the histological examination revealed one lymph node metastasis. She started ARST0121 regimen and radiotherapy with 41.4 Gy for the left neck. Before the initiation of ARST0121 regimen, she has already had radiation retinopathy by the radiotherapy for primary site as a first-line therapy and her visual impairment was not improved in spite of steroid pulse and hyperbaric oxygen therapy. Her visual impairment progressed after the initiation of salvage-line chemotherapy and she decided to stop the chemotherapy after the administration of week 14 therapy of ARST0121 regimen which is originally consisted of the therapy extending to total 50 weeks. She keeps disease free for 5.0 years after the stop of salvage-line therapy.

## Discussion

RMS in adults accounts for fewer than 4% of adult soft tissue sarcoma [2]. Common primary sites of RMS in adults are limbs, head and neck, and genitourinary [12, 13]. Pleomorphic and alveolar types are predominant histology in adult RMS [12, 14]. Alveolar RMS in adults shows more frequencies of nodal and distant metastases compared with embryonal type [13]. The rate of nodal and distant metastases in adult alveolar RMS is 67.6% and 50%, respectively, whereas those in adult embryonal RMS are 19% and 21.7%, respectively. The prognosis of alveolar RMS in adults is poorer than embryonal RMS [13].　The detailed data about kinds of fusion gene in adult alveolar RMS is few. In child alveolar RMS, 85% of patients have fusion gene, of which *PAX3-FOXO1* or *PAX7-FOXO1* are almost all although rare variant fusion genes such as *PAX3-NCOA1* and *PAX3-NCOA2* are reported [15]. In alveolar RMS patients with *PAX* fusion genes, *PAX3-FOXO1* and *PAX7-FOXO1* accounts for 73% and 27%, respectively [15]. The survival outcome in alveolar RMS patients with *PAX* fusion genes are poorer than patients without them [16, 17].

The clinical data of adult PM-HNRMS is still insufficient. Present study reports the characteristics and outcomes of ten adult PM-HNRMS patients who received multi-modal therapy. A previous study on sinonasal RMS in adults reported 5-year OS rates in non-metastatic and metastatic patients of 33.9% and 14.7%, respectively [8]. In the study, about 75% of patients received multi-modal therapy including chemotherapy. In our study, 5-year OS rates in non-metastatic and metastatic patients was 42.8% and 50%, respectively. Oberlin risk factor categories compose of four factors; age, unfavorable site, bone or bone marrow involvement and three or more metastatic sites [18]. The prognosis of patients of metastatic disease with risk factors of zero or one is better than patients with two or more. In the present study, one and three patients in total four metastatic patients had one and two of Oberlin risk categories, respectively. Although the number of patients in our study is small, our study suggests multi-modal therapy including chemotherapy is beneficial for survival in adult PM-HNRMS patients including patients with metastatic diseases.

The prognosis of RMS patients who experience disease relapse or progression even with initial multi-modality therapy is generally poor [19]. Additionally, the survival rate depends on relapse pattern and histological subtype. Distant relapse is associated with worse prognosis than local recurrence and regional lymph node relapse [19]. The prognosis of non-embryonal subtypes is also poorer than that of embryonal subtypes [20]. In a previous study, the 1-year and 2-year OS rates of alveolar subtype RMS after recurrence or relapse were reported to be 40% and 20%, respectively. On the other hand, 1- and 2-year OS rates were 82% and 46%, respectively, in cases with embryonal histology [20]. In addition to the histology, the association of gene mutation status with prognosis is reported [21]. *MyoD1* mutation positive spindle and sclerosing RMS is poor prognosis [22, 23]. The frequency of *MyoD1* mutation is 41–56% in spindle and sclerosing RMS [21, 22, 24]. In consistent with previous reports, the histology of our patient with *MyoD1* mutation positive patient was spindle. In the present study, seven patients including *MyoD1* mutation positive patient had progressive disease after a first-line therapy and two patients progressed during a first-line therapy. Three of seven patient who had progressive disease after or during first-line therapy and received salvage therapy are currently alive. Among them, two patients remained disease-free for over 4 years after completion of the last therapy. Our results suggest that some patients might experience prolonged survival with salvage therapy although the prognosis of RMS patients with relapse is poor. The proposed treatment for RMS in adults recommends the use of multi-modal therapy, with the same protocol as in children but tailored to adults [25, 26]. We treated most patients with a VAC regimen as the first-line therapy. Seven of nine patients started with 2,200 mg/m^2^ of cyclophosphamide, except for a patient aged 63 years and a patient at risk for infection. The median cumulative cyclophosphamide dose was 20.7 g/m^2^ (range 6.6–30.8), which is comparable to that in previous reports [27, 28]. Regarding to vincristine, median administration frequencies was 23 times compared with about maximum 30 times. Relative dose intensity of vincristine is about 77%, which is less than the 90.2% in children [27]. This decrease of dose intensity was mainly due to peripheral neuropathy. The management of neurotoxicity is one of important issues in adult RMS when conducting treatment based on pediatric protocol. There was no therapy-related death. Regarding salvage chemotherapy, a standard regimen has, so far, not been established [29]. The efficacy of high-dose chemotherapy followed by autologous stem cell transplantation is also not established in RMS including salvage-line in adults [30, 31].　Randomized trial comparing high dose chemotherapy followed by autologous stem cell transplantation with standard chemotherapy is expected. The two patients who remained disease-free after the completion of salvage therapy in our study received the ARST0121 regimen as salvage chemotherapy [11]. Previous studies indicated that the prognosis of patients who could be treated with adequate local therapy combined with chemotherapy was better than those who could not receive such therapy [32]. Other studies also showed the benefits of surgery and/or radiation in addition to chemotherapy in suitable cases of recurrence and/or relapsed RMS [32, 33]. The two patients who remained disease-free after the completion of salvage therapy in our study also received local therapy for all detected lesions.

The development of new therapies for RMS is expected. Temsirolimus, the inhibitor of mammalian target of rapamycin (mTOR), showed the efficacy for relapsed RMS in comparison with bevacizumab when combined with vinorelbine and cyclophosphamide in phase II trial [34]. In the preclinical study, CRISPR-interference screen identified the dependency of *PAX3-FOXO1* positive RMS on *GATOR2* complex which is the positive regulator of mTORC1 dependent on amino acid [35].　ARST1431, phase III trial, is ongoing to investigate the efficacy of the addition of temsirolimus on chemotherapy [36]. Transcriptional architecture of rhabdomyosarcoma requires the balanced status of histone modification [37].　The utility of epigenetic modifiers and chromatin remodelers such as histone deacetylase (HDAC) inhibitors and bromodomain and extraterminal domain (BET) protein inhibitor, respectively, are under investigation [38, 39].　Genomic landscape has been proven in RMS. *NRAS*/*KRAS*/*HRAS*, *FGFR4*, *NF1*, *PIK3CA* an *BCOR* mutations occur mainly in *PAX* fusion negative RMS [15, 23]. On the other hand, *MDM2* amplification without *TP53* alteration is detected in *PAX* fusion positive RMS. *CDK4* and *MYCN* amplification is also reported mainly in *PAX* fusion positive RMS. In *MyoD1*-mutant RMS, co-mutation of *PIK3CA* and/or *CDKN2A* is reported. Targeted therapies dependent on these molecular alterations are expected [40].

The present study has several limitations. First, the sample size was small because of the rarity of adult PM-HNRMS cases. Second, heterogenous regimens were used for salvage chemotherapy in the present study. Therefore, it is difficult to compare the clinical outcome between patients receiving each regimen of salvage chemotherapy.

## Conclusions

We presented the clinical outcomes of adult PM-HNRMS patients. The cure rate of adult PM-HNRMS is low in spite of a first-line therapy. Salvage therapy might prolong the survival in patients who received the multi-modal therapy including local therapy for all detected lesions.

## Data Availability

The datasets used and/or analysed during the current study available from the corresponding author on reasonable request.

## Abbreviations

RMS: Rhabdomyosarcoma
HNMRS: Head and neck rhabdomyosarcoma
PM: Para-meningeal
IRS: The Intergroup Rhabdomyosarcoma Study
OS: Overall survival
VAC: Vincristine, actinomycin D and cyclophosphamide
VI: Vincristine and irinotecan
VDC/IE: Vincristine, doxorubicin and cyclophosphamide/ifosfamide and etoposide
DC: Doxorubicin and cyclophosphamide
ICE: Ifosfamide, carboplatin and etoposide
CT: Computed tomography
PFS: Progression-free survival
RECIST: The Response Evaluation Criteria in Solid Tumors
COG: The Children’s Oncology Group
NR: Not reached
